# Evolution and Developmental System Drift in the Endoderm Gene Regulatory Network of *Caenorhabditis* and Other Nematodes

**DOI:** 10.3389/fcell.2020.00170

**Published:** 2020-03-18

**Authors:** Chee Kiang Ewe, Yamila N. Torres Cleuren, Joel H. Rothman

**Affiliations:** ^1^Department of Molecular, Cellular, and Developmental Biology, University of California, Santa Barbara, Santa Barbara, CA, United States; ^2^Neuroscience Research Institute, University of California, Santa Barbara, Santa Barbara, CA, United States; ^3^Computational Biology Unit, Department of Informatics, University of Bergen, Bergen, Norway; ^4^Department of Ecology, Evolution, and Marine Biology, University of California, Santa Barbara, Santa Barbara, CA, United States

**Keywords:** *Caenorhabditis*, developmental system drift, developmental hourglass, plasticity, robustness

## Abstract

Developmental gene regulatory networks (GRNs) underpin metazoan embryogenesis and have undergone substantial modification to generate the tremendous variety of animal forms present on Earth today. The nematode *Caenorhabditis elegans* has been a central model for advancing many important discoveries in fundamental mechanistic biology and, more recently, has provided a strong base from which to explore the evolutionary diversification of GRN architecture and developmental processes in other species. In this short review, we will focus on evolutionary diversification of the GRN for the most ancient of the embryonic germ layers, the endoderm. Early embryogenesis diverges considerably across the phylum Nematoda. Notably, while some species deploy regulative development, more derived species, such as *C. elegans*, exhibit largely mosaic modes of embryogenesis. Despite the relatively similar morphology of the nematode gut across species, widespread variation has been observed in the signaling inputs that initiate the endoderm GRN, an exemplar of developmental system drift (DSD). We will explore how genetic variation in the endoderm GRN helps to drive DSD at both inter- and intraspecies levels, thereby resulting in a robust developmental system. Comparative studies using divergent nematodes promise to unveil the genetic mechanisms controlling developmental plasticity and provide a paradigm for the principles governing evolutionary modification of an embryonic GRN.

## Introduction

From the moment of fertilization, embryos must follow a highly regulated script that ensures reproducible outcomes, while remaining plastic to accommodate changes that generate morphological diversity. The architectures of the gene regulatory networks (GRNs) are sculpted by, and can greatly influence, evolutionary trajectory, raising central questions in evolutionary developmental biology. How are networks wired in ways that ensure developmental robustness? Which nodes are plastic and which nodes are more rigidly fixed?

The endoderm is the most ancient of the three germ layers in animals and the GATA-driven core regulatory pathway that directs endoderm development is conserved across metazoans, including in the most basal diploblastic animals (Rodaway and Patient, 2001; Martindale et al., 2004; Hashimshony et al., 2015). Thus, understanding the mechanisms that deploy the endoderm GRN is critical to revealing the fundamentals of cell fate acquisition and body plan organization during animal embryogenesis. This brief review will examine evolutionary diversification of the GRN in nematodes. We will discuss developmental system drift (DSD) at both micro- and macroevolutionary scales. Finally, we will visit the developmental hourglass model in relation to endoderm development.

## Nematodes as Models for Investigating Evolutionary Diversification of the Endoderm GRN

Since Sydney Brenner first introduced a free-living roundworm, *Caenorhabditis elegan*s, to the broad research community, the domesticated laboratory strain, obtained from Bristol, England and named “N2,” has become a key contributor to many important discoveries in developmental, cellular, and molecular biology, owing to its ease of propagation, ready access to genetic analysis, and the plethora of resources available for it, including the complete description of its anatomy (White et al., 1986; Cook et al., 2019) and cell lineage (Sulston and Horvitz, 1977; Sulston et al., 1980, 1983). The potent molecular genetic toolkits available with the system has resulted in identification of a large collection of mutants that have allowed experimentalists to dissect the mechanistic processes that orchestrate development in the animal (Thompson et al., 2013). These discoveries have provided a springboard for understanding the evolutionary steps that result in diversification of developmental mechanisms.

Two major strategies have been taken to investigate evolutionary variation in nematode developmental mechanisms: studies on representative species that span nematode phylogeny (Sommer and Bumbarger, 2012; Haag et al., 2018), and analysis of evolutionarily divergent isolates of one species, *C. elegans* (Barrière and Félix, 2005). The former strategy has involved both comparative embryology and phylogenomics studies of nematodes arising from distinct clades, revealing deeper changes in both developmental and gene regulatory strategies. These studies have taken advantage of the >50 species spanning the *Caenorhabditis* genus that have been isolated as well as nematodes from distant clades (1–12), many of which have had their genomes sequenced (Holterman et al., 2006; Kiontke et al., 2011; Sommer and Streit, 2011; Schiffer et al., 2013; Félix et al., 2014; Ferrari et al., 2017; Hiraki et al., 2017; Stevens et al., 2019). In contrast, insights into how endoderm regulatory events occur over shorter evolutionary time-frames have provided a better understanding of how specific steps in the endoderm GRN are tuned during radiation of a species. These latter studies have been facilitated through quantitative genetics approaches, by availing of over 300 wild isolates obtained from different continents over the past decade, whose genomes have been fully sequenced (Andersen et al., 2012; Cook et al., 2016, 2017; Zhao et al., 2018). A recent sampling effort in Hawaii further identified *C. elegans* strains that show large amount of genetic diversity (Crombie et al., 2019). These rich resources offer a unique and attractive opportunity for both intra- and interspecies comparative studies.

## The *C. elegans* Endoderm Gene Regulatory Network

**FIGURE 1:**
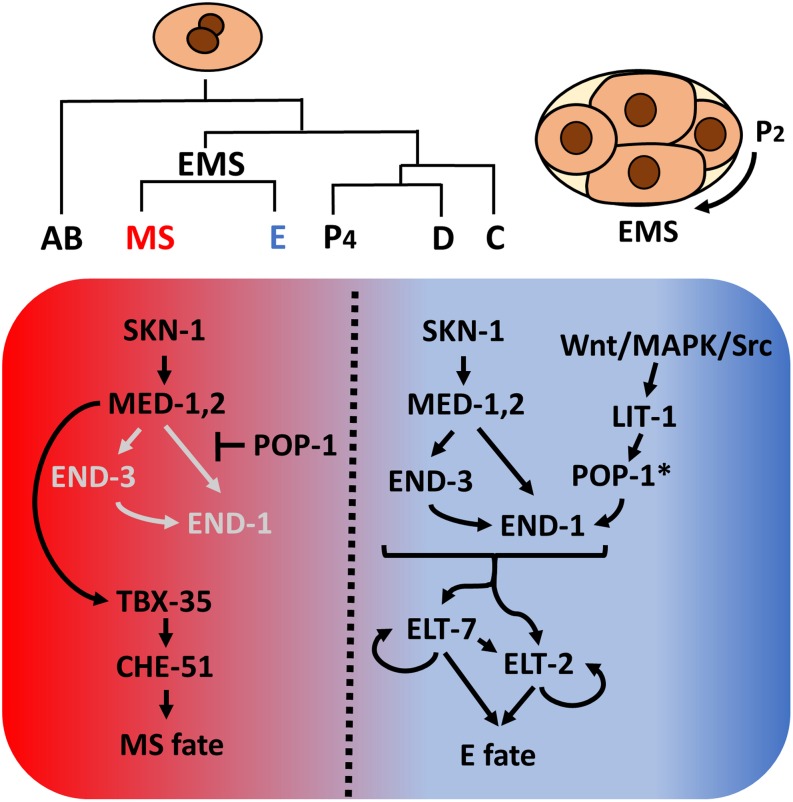
*Caenorhabditis elegans* founder cells and the endoderm specification network. Asymmetrical cell divisions produce six founder cells, each of which will give rise to specific tissue types. At the four-cell stage, SKN-1 activates the *med-1,2* genes, initiating mesendoderm specification. Redundant Wnt/MAPK/Src signaling arising from the neighboring P_2_ cell polarizes EMS. In the anterior, un-signaled end, POP-1 represses *end-1* and *end-3* expression while MED-1,2 turn on *tbx-35*, which in turn specify mesoderm MS fate. In the posterior end, LIT-1 kinase, in response to P_2_ signals, phosphorylates POP-1 (indicated by *), converting it from a repressor to an activator of endoderm E fate. The two differentiation factors, ELT-7 and ELT-2, once activated, maintain their own expression through autoregulation and regulate thousands of gut genes. In E, Wnt signaling further represses *tbx-35* expression (Broitman-Maduro et al., 2006).

As was first recognized with the nematode *Ascaris megalocephala* by Theodor Boveri over a century ago (Boveri, 1899), early *C. elegans* embryogenesis is essentially invariant, resulting in generation of six founder cells (AB, MS, E, C, D, and P_4_) through a series of asymmetrical cleavages. As is the case with most other nematodes (Malakhov, 1994; Wiegner and Schierenberg, 1998; Houthoofd et al., 2003; Zhao et al., 2008; Schulze and Schierenberg, 2011), the entire *C. elegans* intestine, is derived clonally from the E blastomere (Sulston et al., 1983), providing a highly tractable system to study cell specification, differentiation and organogenesis. Studies over the past three decades have provided a high-resolution description of the endoderm GRN (reviewed in Maduro and Rothman, 2002; McGhee, 2007; Maduro, 2015, 2017). In brief, maternally provided SKN-1/Nrf activates a zygotically expressed transcriptional cascade comprising a series of GATA-like transcription factors, including the GATA-like factors MED (MesEnDoderm)-1 and MED-2, which bind to a non-canonical RRRAGTATAC site (Broitman-Maduro et al., 2005), and the canonical GATA factors END-3 and END-1. This leads to the activation of ELT-7 and ELT-2, which, together, drive activation of thousands of gut-expressed genes and morphological differentiation of the intestine (Fukushige et al., 1998; McGhee et al., 2009; Sommermann et al., 2010; Dineen et al., 2018). SKN-1 and MED-1/2 also function in the sister of E, MS, to activate mesoderm development (Figure 1).

Endoderm fate is activated by an inductive cellular interaction: a triply redundant Wnt/MAPK/Src signaling system triggered by signals from the neighboring P_2_ cell polarizes the mesendodermal EMS cell and subsequently modifies the nucleocytoplasmic distribution and activity of POP-1/Tcf (Maduro et al., 2002; Shetty et al., 2005; Phillips et al., 2007; Owraghi et al., 2010). In the un-signaled MS cell, POP-1 represses *end-1* and *-3* expression, thereby inhibiting gut fate. In the posterior E cell, the inductive signal results in phosphorylation of POP-1 by LIT-1/Nlk, converting it from a repressor to an activator of E fate. Thus, SKN-1 and POP-1 play a partially redundant role in endoderm specification in *C. elegans* (Figure 1). In recent years, mutant/RNAi screens, proteomic, and transcriptomic studies revealed many novel regulators implied in endoderm development and embryogenesis (Witze et al., 2009; Du et al., 2014; Sullivan-Brown et al., 2016; Tintori et al., 2016; Dineen et al., 2018; Wiesenfahrt et al., 2018). The elucidation of *C. elegans* endoderm GRN provides a strong foundation from which to explore the diversification of endoderm GRN in other organisms.

## Variation in Endoderm Developmental Strategies

Closely related species in the Elegans supergroup show nearly identical cell lineages to those of *C. elegans* (Zhao et al., 2008; Levin et al., 2012). Similarly, *Pristionchus pacificus*, which belongs to clade 9, along with the *Caenorhabditi*s species, shows a similar pattern of early embryonic division, differing mostly in cell cycle timing (Vangestel et al., 2008). Nevertheless, it has been shown that early embryonic development is highly divergent in Nematoda, especially in the basal Enoplae (clades 1 and 2). For example, in *Enoplus brevis*, only the E lineage is specified in the very early embryo, while the remaining cells become committed later at the 30–60 cell stage (Voronov and Panchin, 1998; Schulze and Schierenberg, 2011). In contrast to Enoplea, perhaps with the exception of *Romanomermis culicivorax* (clade 2) (Schulze and Schierenberg, 2009), Chromadorea (clades 3–12) contains largely defined cell lineages during early embryogenesis, transitioning from a “regulative” to a more or less “mosaic” pattern of development, although the organization of the founder cells may vary (Dolinski et al., 2001). In the case of *Acrobeloides nanus* (clade 11), and in sharp contrast to the Elegans group, the founder cells remain multipotent: EMS can become AB, and C can replace EMS at the three-cell stage. Furthermore, unlike in *C. elegans*, which requires inductive interactions between EMS and P_2_ cells, endoderm specification in *A. nanus* appears to occur cell-autonomously, such that isolated EMS, AB, or P_2_ can give rise to differentiated gut cells and the restriction of cell fate instead depends on the inhibitory interactions between the blastomeres (Wiegner and Schierenberg, 1998, 1999).

**FIGURE 2:**
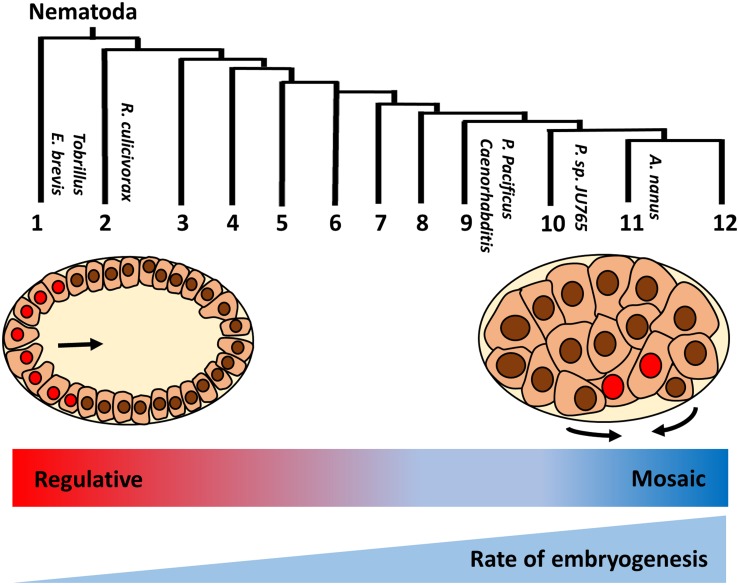
Variation in early embryogenesis in Nematoda. Nematodes are classified into 12 clades based on rDNA sequence (Holterman et al., 2006). Basal *Tobrillus* undergoes a “canonical” protostome-like gastrulation characterized by invagination of eight endoderm precursors (red nuclei) at the anterior blastopore during 64 cell-stage. Gastrulation in more highly derived nematodes is driven by apical constriction of endoderm precursors at the postero-ventral surface of 28 cell-stage embryo (adapted from Joshi and Rothman, 2005). Unlike species in the early branching clades, in which cell fates are plastic and rely on external signals (“regulative” development), cell lineages are largely fixed during early division (“mosaic development”) in more derived species. In addition, developmental rate is faster in the more derived clades. Thus, it is proposed that heterochronic and heterotopic shift in the developmental program drive the evolution of early embryogenesis in nematodes.

While gastrulation in many clades is initiated by the inward movement of two endoderm progenitors on the ventral posterior side of the early embryo following division of the E founder cell, this appears to be a highly derived characteristic that is not typical for protostomes. Interestingly, a basal freshwater nematode, *Tobrilus* (clade 1), undergoes gastrulation marked by the presence of a large blastocoel and the anterior invagination of endo- and mesodermal precursors (Schierenberg, 2005; Schulze and Schierenberg, 2011; Figure 2). This gastrulation process resembles the classical protostome pattern, in which a collection of cells invaginate at a blastopore that is the future site of the mouth. It should be noted that this is not an inviolable characteristic of protostomes: in some Ecdysozoans, including in Nematomorpha, the sister taxa of Nematoda, gastrulation resembles that of deuterostomes, in which the blastopore forms at the future site of the anus (Montgomery, 1904; Martín-Durán et al., 2012). The invention of the highly derived “phylotypic” pattern of gastrulation seen in *C. elegans* and in most nematodes, and the transition of a “regulative” to a “mosaic” mechanism of cell fate specification, generally correlate with embryos that undergo rapid development. It is tempting to postulate that increasing reliance on maternal factors during evolution allows for rapid cell cycle and cell specification during early embryogenesis (Wiegner and Schierenberg, 1998; Laugsch and Schierenberg, 2004). This may result in heterochronic (timing) and heterotopic (spatial) shift in the developmental program, leading to the different modes of specification and gastrulation (Figure 2; Joshi and Rothman, 2005).

## Evolution of the Endoderm GRN in Nematodes

While orthologs of SKN-1, which is essential for initiating mesendoderm specification in *C. elegans*, are found across divergent nematode species, its action in endoderm development varies dramatically between them. Maternally provided *skn-1* RNA is initially present throughout the *C. elegans* early embryo but becomes differentially lost in somatic blastomeres and is maintained in the germline lineage (Seydoux and Fire, 1994). In contrast, a very different pattern is observed in *Propanagrolaimus* sp. JU765 (clade 10) and *A. nanus*, in which *skn-1* mRNAs, which are presumably zygotic products, accumulate in all somatic blastomeres through much of embryogenesis (Wiegner and Schierenberg, 1998; Schiffer et al., 2014, 2018). These observations suggest differential regulation of *skn-1* expression and that, in addition to activating mesendoderm specification, SKN-1 may perform distinct functions in species from neighboring clades. Remarkably, the requirement for SKN-1 in endoderm specification varies even in closely related *Caenorhabditis* species. In *C. elegans*, eliminating SKN-1 results in a partial penetrant loss-of-endoderm phenotype, as SKN-1 and POP-1 function through an “OR” Boolean logic gate (Figure 1). However, in *Caenorhabditis briggsae*, which diverged from *C. elegans* ∼20–40 million years ago, both SKN-1 and POP-1 show an absolute requirement in endoderm specification, indicative of an “AND” logic gate (Cutter, 2008; Lin et al., 2009). These observations suggest that the early inputs into the endoderm GRN are rapidly evolving in nematodes.

The gut terminal differentiation factors, including ELT-2/GATA and the FoxO factor PHA-4 are conserved across Nematoda (Schiffer et al., 2014; Maduro, 2020). In contrast, the upstream *med* and *end* orthologs are present only in closely related *Caenorhabditis* species, apparently having arisen as a result of extensive gene duplication events at the base of the Elegans supergroup, as revealed in a recent study that examined the evolutionary variation in the GATA regulatory cascade across 24 species spanning the *Caenorhabditis* genus (Maduro, 2020). In two strikingly extreme cases, *Caenorhabditis doughertyi* and *Caenorhabditis brenneri* each contain ∼30 copies of the *med* genes (Maduro, 2020). This massive proliferation of protein-coding genes is highly unusual and may reflect adoption of new functions by at least some of the paralogs, as exemplified by the expansion of another class of transcription factors in *Caenorhabditis* species, the nuclear hormone receptors (NHRs) (Taubert et al., 2011). Most *Caenorhabditis* NHRs appear to have arisen from an ancestral Hepatocyte Nuclear Factor 4 (HNF4)-type NHR and appear to have evolved to perform diverse roles ranging from neural development (Zhou and Walthall, 1998; Much et al., 2000) to metabolic control (Gilst et al., 2005; Wang et al., 2015) to sex determination (Ilil et al., 1998). It is conceivable that changes in the *cis*-regulatory regions lead to differential expression and subsequent functional divergence of the MED paralogs, leading to retention of gene duplicates (True and Haag, 2001; Gissendanner et al., 2004; Taubert et al., 2011), though the function of MEDs beyond mesendoderm development have not been described. Importantly, functional diversification of duplicate genes can also drive rapid changes in developmental programs and DSD (True and Haag, 2001; Haag et al., 2018). For example, the *C. briggsae* translational regulator PUF (PUmilio and FBF)*-*2 plays a non-redundant role in pharynx and vulva development, in addition to promoting gametogenesis, the sole known role of its paralog PUF-1.2 and its homologs in *C. elegans* (Liu et al., 2012; Liu and Haag, 2014). Although morphologically invariant, the molecular mechanisms underlying vulva development vary across nematodes (Sommer and Sternberg, 1994; Félix et al., 2000; Dichtel-Danjoy and Félix, 2004; Zheng et al., 2005; Félix, 2007), which may, at least partly, have been caused by DSD resulting from gene duplication.

What might account for the expansion of GATA factors in the *Caenorhabditis* endoderm GRN? The cascade of redundant factors may function to ensure developmental robustness during the rapid embryogenesis characteristic of this clade. In *C. elegans*, and likely in the other *Caenorhabditis* species (Wiesenfahrt et al., 2016; Maduro, 2020), the endoderm GATA factors form recursive feedforward loops, which may provide a rapid, forward-driven activation switch. In addition, the small size of the MEDs (174 residues) and ENDs (221–242 residues), compared to ELT-2 (433 residues) and SKN-1 (∼600 residues), may allow for more rapid deployment of the cascade and lockdown of gut fate, perhaps owing to more rapid synthesis and access to chromatin (Maeshima et al., 2015). Another potential explanation is that the GATA cascade may allow more robust expression of ELT-2. The provision of maternal factors can vary among individuals (Nuzhdin et al., 2008; Surkova et al., 2008; Perez et al., 2017) especially under conditions of environmental stress, which is mitigated by SKN-1 (An and Blackwell, 2003; Crofton et al., 2018; Jordan et al., 2019). Intercession of the MEDs and ENDs in the cascade may therefore free *elt-2* from direct control of SKN-1, thereby buffering against changes in environmental conditions. Finally, redundancy in the system allows for evolutionary experimentation and accumulation of cryptic genetic variants, promoting the evolution of the system (Félix and Wagner, 2008) (see below).

## Rapid Developmental System Drift Among *C. elegans* Wild Isolates

Most of our understanding of *C. elegans* biology is based on studies on a single genetic background, that of the laboratory reference strain N2. The identification of wild *C. elegans* isolates bearing distinct haplotypes has uncovered considerable phenotypic variation and developmental plasticity in this species (Hodgkin and Doniach, 1997; Harvey et al., 2008; Milloz et al., 2008; Andersen et al., 2012; Alcorn et al., 2016; Cook et al., 2016; Greene et al., 2016; Frézal et al., 2018; Gimond et al., 2019; Lee et al., 2019). Knocking down essential genes in the wild strains yielded distinct phenotypes and has uncovered substantial cryptic variation between the spectrum of isotypes (Paaby et al., 2015; Torres Cleuren et al., 2019). In addition, while the overall morphology remains constant, the network architecture underlying vulva induction is variable in wild genetic backgrounds (Milloz et al., 2008; Duveau and Félix, 2012). Environmental cues can modulate activities in the vulva signaling network and the sensitivity of the system varies among divergent *C. elegans* isotypes (Braendle and Félix, 2008; Grimbert and Braendle, 2014). Thus, potential incipient changes in developmental regulatory networks, and their robustness to environmental variation, can be revealed by examining the requirement for components in the networks in genetically distinct wild isolates.

A recent study uncovered striking variation in the endoderm GRN among the wild isolates, as reflected by the differential requirement of maternal SKN-1 and the endoderm-inducing MOM-2/Wnt (Torres Cleuren et al., 2019). This study revealed in part that the two activating pathways exhibit a partially compensatory relationship, in which a weaker requirement for the SKN-1 input is accompanied by a stronger requirement for the MOM-2 input and *vice-versa*, which may tune the levels of the activating signals to ensure a constant developmental outcome (Maduro et al., 2015; Choi et al., 2017; Torres Cleuren et al., 2019). Thus, the accumulation of cryptic genetic variants drives rewiring of the inputs into the endoderm GRN. This rapid DSD may be the result of the extensive redundancy in the system, which permits cryptic genetic variants to arise without diminishing fitness, and allows compensatory evolution to occur.

What are the genetic mechanisms governing plasticity in the endoderm GRN? How do the endoderm regulatory inputs respond to environmental perturbation? How do the *cis*-regulatory elements of endoderm genes differ between wild isolates? Using quantitative genetic methods coupled with molecular tools, the even-expanding collection *C. elegans* isotypes provides a powerful platform for dissecting the evolution of complex traits and the assembly of GRNs (Cook et al., 2017).

## Developmental Hourglass Model: Plasticity and Conservation of Endoderm Development

As discussed above, the early inputs into the endoderm GRN are highly variable across nematodes and show dramatic plasticity even within a single species. This is in accordance with the hourglass model of embryonic development, in which divergent developmental mechanisms converge on a phylotypic stage, which may coincide with expression of conserved differentiation factors (Duboule, 1994; Raff, 1996). Comparisons of early embryonic transcripts across many *Drosophila* species and the mosquito *Aedes aegypti* has revealed that maternal transcript pools that, like those of *C. elegans skn-1*, are present only transiently during early embryogenesis, and that their expression levels are highly variable across these species, spanning ∼60 million years of evolution (Atallah and Lott, 2018). Similarly, considerable variation in the expression of maternal factor genes is found between different nematode species (Levin et al., 2012; Macchietto et al., 2017; Schiffer et al., 2018). This hourglass pattern of variation is attributable to the lack of negative selection of maternal-effect genes (Barker et al., 2005; Cruickshank and Wade, 2008; Cutter et al., 2019), as well as to increased developmental constraints during mid-embryogenesis (Raff, 1996; Zalts and Yanai, 2017). It will be of interest to ask whether the variation in SKN-1 dependence between *C. briggsae* and *C. elegans* isotypes results from quantitative changes in *skn-1* expression and/or alteration of the *cis*-regulatory sites of its targets (Peter and Davidson, 2011; Verster et al., 2014; Vu et al., 2015). Indeed, the number of putative SKN-1 binding sites in *end-3* and *end-1* promoters has been found to vary widely, and in some cases the sites are absent or unrecognizable, in many *Caenorhabditis* species (Maduro, 2020).

It has been shown that, relative to early and late embryonic development, gene expression during morphogenesis is highly conserved, not only across *Caenorhabditis*, but also across Bilateria (Levin et al., 2012). Cellular patterning during mid-embryogenesis is similar in nematodes from distant clades, despite extensive variation in early division (Schulze and Schierenberg, 2011). In many nematode species, the endodermal daughters migrate from the ventral side into the interior of the embryo during gastrulation, as in *C. elegans* (Figure 2; Vangestel et al., 2008; Schulze and Schierenberg, 2011; Schulze et al., 2012; Calderón-Urrea et al., 2016). This is followed by proliferation and polarization of the intestine primordia, and subsequent formation of lumen through cell rearrangements and remodeling, similar to gut morphogenesis observed in zebrafish (reviewed in Nowotschin et al., 2019). The action of ELT-2 (and ELT-7) at the end of the endoderm cascade, where they act on thousands of targets that underlie morphological differentiation and function of the gut, presumably restricts evolutionary divergence at this node, whereas the earlier nodes of the GRN involve the action of transcription factors with far fewer target genes, hence allowing for much greater evolutionary plasticity (see also Maduro and Rothman, 2002).

## Conclusion

With the molecular details of the *C. elegans* endoderm GRN in hand, the mechanisms that govern the diversification of the network in other nematode species have begun to emerge. For example, while the endoderm fate is confined to a single cell in *C. elegans*, all blastomeres are potentiated to become gut and cell fate is regulated through lateral inhibition in *A. nanus*. Comparing the mechanisms of cell fate restriction between these species will not only enhance our understanding of evolutionary plasticity and reprogramming of GRNs, but also provide important insights into how such a system transitions from one configuration into another during evolution. One curious element of the endoderm GRN is that there appears to be substantial cross-talk between some endoderm components and stress response pathways (An and Blackwell, 2003; Wheeler and Thomas, 2006; Arsenovic et al., 2012; Block et al., 2015; Dresen et al., 2015; Ewe et al., 2019). Pleiotropic genes modulating stress pathways may act cryptically in endoderm development. Changes in environmental conditions may then lead to selection and fixation of cryptic variants, resulting in rapid DSD (Johnson and Porter, 2007; Duveau and Félix, 2012). By availing of nematodes isolated from diverse geographical locations, including those from extreme habitats (Shih et al., 2019), it will be of interest to ask how environment cues shape the endoderm GRN structure.

The mechanisms of early specification of gut fate appear to undergo rapid and widespread changes at both inter- and intraspecies levels. This unexpectedly high degree of evolutionary plasticity in the system that establishes the most ancient germ layer can serve as an excellent paradigm for DSD. The development of molecular genetic tools that can be applied to nematodes outside of *C. elegans* (Lok and Unnasch, 2007; Wood et al., 2011; Lo et al., 2013; Kanzaki et al., 2018; Cohen and Sternberg, 2019), together with new sequencing technologies that integrate multi-omic analyses (Witze et al., 2009; Daugherty et al., 2017; Guo et al., 2017; Packer et al., 2019), will greatly facilitate the study of complex developmental regulatory systems and their evolutionary trajectory across divergent species.

## Author Contributions

CE and YT wrote the first draft of the manuscript. JR directed the project and contributed to the manuscript revisions. All authors approved the submitted version.

## Conflict of Interest

The authors declare that the research was conducted in the absence of any commercial or financial relationships that could be construed as a potential conflict of interest.
